# Genetic Polymorphisms in the *HMGCR* Gene and Associations with Cognitive Decline in Parkinson’s Disease Patients

**DOI:** 10.3390/ijms25168964

**Published:** 2024-08-17

**Authors:** Anna Pierzchlińska, Jarosław Sławek, Magdalena Kwaśniak-Butowska, Damian Malinowski, Nina Komaniecka, Monika Mak, Anna Czerkawska, Arnold Kukowka, Monika Białecka

**Affiliations:** 1 Department of Pharmacokinetics and Therapeutic Drug Monitoring, Pomeranian Medical University, 70-111 Szczecin, Poland; pierzchlinska@gmail.com (A.P.); ania.czerkawska@wp.pl (A.C.); arnold.kukowka@gmail.com (A.K.); monika-bialecka@post.pl (M.B.); 2 Department of Animal Physiology, Institute of Zoology, University of Cologne, 50923 Cologne, Germany; 3 Department of Neurological-Psychiatric Nursing, Faculty of Health Sciences, Medical University of Gdańsk, 80-211 Gdańsk, Poland; jaroslaw.slawek@gumed.edu.pl (J.S.); magda.kwasniak@gmail.com (M.K.-B.); 4 Department of Neurology, St Adalbert Hospital, 61-144 Gdańsk, Poland; 5 Department of Experimental and Clinical Pharmacology, Pomeranian Medical University, 70-111 Szczecin, Poland; nina.komaniecka@pum.edu.pl; 6 Department of Health Psychology, Pomeranian Medical University, 70-111 Szczecin, Poland; monika.mak@pum.edu.pl

**Keywords:** Parkinson’s disease, HMGCR, dementia, cognitive decline, genetic polymorphisms, lipids

## Abstract

Parkinson’s disease (PD) is a common neurodegenerative disease characterized by motor and non-motor symptoms including cognitive impairment and dementia. The etiopathogenesis of PD, as well as its protective and susceptibility factors, are still elusive. 3-Hydroxy-3-methyglutaryl coenzyme A reductase (HMGCR) is an enzyme regulating cholesterol synthesis. Single-nucleotide polymorphisms (SNPs) in the gene coding *HMGCR* have recently been correlated with the risk of Alzheimer’s disease. Alternative splicing of exon 13 of the *HMGCR* transcript and its strongly associated *HMGCR* haplotype 7 (H7: rs17244841, rs3846662, rs17238540) may downregulate protein activity and cholesterol synthesis, with lower low-density lipoprotein cholesterol (LDL) levels associated with PD that may affect cognitive abilities. We genotyped three SNPs in the H7 *HMGCR* gene in 306 PD patients divided into three groups—without cognitive decline, with mild cognitive impairment (MCI), and with PD dementia—and in 242 healthy participants. A correlation between the rs17238540 genotype and PD susceptibility as well as a minor association between rs3846662 and cognitive status in PD patients was observed; however, the two-sided analysis of these groups did not reveal any significance. We observed a statistically significant elevated high-density lipoprotein cholesterol (HDL) plasma level in the minor allele carriers of rs17238540 and rs17244841 among PD patients. This study should be replicated in a larger population.

## 1. Introduction

Parkinson’s disease (PD) is a neurodegenerative disease affecting neurons mostly in the substantia nigra pars compacta (SNpC) area, which results in progressive dopamine deficiency in the basal ganglia and associated motor symptoms, e.g., bradykinesia, resting tremor, rigidity, or gait disturbance. However, numerous non-motor features have been described, including sleep disturbance, autonomic dysfunction, depression, and cognitive impairment, with dementia affecting up to 75% of PD patients [1,2]. The motor symptoms of PD are the consequence of the loss of dopaminergic (DA) neurons within the substantia nigra (SN), although other neurotransmitter systems (i.e., glutamatergic, cholinergic, tryptaminergic, noradrenergic, adrenergic, serotoninergic, and peptidergic) also appear to be affected. Although the pathological changes contributing to the disease have been thoroughly analyzed, i.e., α-synuclein aggregations in the form of Lewy bodies and Lewy neurites [3,4], the exact mechanisms leading to the occurrence of pathological changes are still unclear. A certain section of the investigations towards the underlying mechanism focuses on the possible effects of metabolic dysregulation, e.g., in the lipid metabolism [5].

The brain is the most cholesterol-rich organ of the human body. Cholesterol builds the myelin sheath and plasma membranes of neurons and astrocytes [6]; thus, it is essential for the functioning of the central nervous system. On the other hand, the impact of cholesterol and its metabolites on oxidative stress and neuroinflammation is well studied. Cholesterol has been associated with α-synuclein, β-amyloid aggregation, and dopaminergic cell degeneration [7]. Moreover, high cholesterol levels determine metabolic syndrome, which increases the risk of mild cognitive impairment (MCI) and dementia in PD (PDD) [8]. To date, the relationship between high total cholesterol and PD remains unclear [9,10,11].

Medical compounds that inhibit cholesterol synthesis by acting on 3-hydroxy-3-methyl-glutarylcoenzyme A (HMG-CoA) reductase—statins—exhibit antioxidant, anti-inflammatory, and anti-excitotoxic properties in vitro, suggesting their neuroprotective role [12]. However, research on their effects on PD in human subjects has shown contradictory results [13]. Apart from methodological reasons, these differences may result from the genetic variability of the HMG-CoA reductase’s gene *HMGCR*. Single-nucleotide polymorphisms (SNPs: rs17244841, rs3846662, and rs17238540) forming a haplotype (H7), one of the many alternative splice variants of exon 13 of the *HMGCR* transcript, were shown to cause defects in the substrate-binding domain of the enzyme and decreased low-density lipoprotein (LDL) cholesterol reduction in statin-treated patients [14,15,16]. On the other hand, HMG-CoA reductase constitutes the rate-limiting enzyme of cholesterol synthesis; thus, changes in its gene can shift the balance of cholesterol in the brain and on the periphery, alternating the risk of neurodegeneration. One of the H7 SNPs, rs3846662, was found to influence the risk of Alzheimer’s disease (AD), age of onset, and risk of conversion of MCI to AD [17].

The aim of the present study was to examine whether the three rs17238540, rs17244841, and rs3846662 polymorphisms in the *HMGCR* gene are related to PD susceptibility, cognitive impairment (MCI), or dementia in PD patients.

## 2. Results

The PD-NCI (Parkinson’s disease patients without cognitive impairment), PD-MCI (Parkinson’s disease patients with mild cognitive impairment), and PDD (Parkinson’s disease dementia patients) groups did not reveal any significant associations in terms of sex (*p* = 0.593) but varied significantly in the mean age of the participants (*p* < 0.001), disease duration (*p* = 0.002), age at disease onset (*p* < 0.001), UPDRS (Unified Parkinson’s Disease Rating Scale) score (*p* < 0.001), and daily L-dopa dosage (*p* = 0.0005), with the highest mean values for all of them in the PDD group (Table 1). The mean age of the study group did not differ from that of the control group (*p* = 0.261). Groups varied in terms of sex proportions, as in the control group, the female sex was dominant (*p* < 0.001).

We did not find any significant differences between the PD and control groups in *HMGCR* rs17244841, rs3846662, and rs17238540 genotypes (*p* = 0.252, *p* = 0.302, and *p* = 0.611, respectively, Table 2). The genotype distributions of all tested polymorphisms met the Hardy–Weinberg equilibrium, except rs17238540: in both the analyzed groups, the number of rs17238540 heterozygous carriers (GT) was expected to be higher, while the numbers of the homozygous carriers were expected to be lower according to the χ2 test calculations (GG and TT genotypes).

The genotype and allele frequencies of the analyzed polymorphisms in the PD group did not vary significantly for HMGCR rs17244841 and rs17238540 (Table 3). However, we did find associations between the alleles of rs3846662; the major AA genotype was observed more frequently with increasing cognitive impairment: PD-NCI 23.5% vs. PD-MCI 29.5% vs. PDD 40%, *p* = 0.041.

Comparisons of the genotype and allele distributions between the analyzed groups were as follows: (1) PD-NCI vs. PD-MCI  +  PDD, (2) PD-NCI  +  PD-MCI vs. PDD, (3) PD-NCI vs. PD-MCI, and (4) PD-NCI vs. PDD did not reveal any significant differences (data not presented).

A statistical analysis of the patient data was conducted to identify any differences in lipid levels in regard to the *HMGCR* genotype. However, no significant differences were observed in lipid levels and body mass index (BMI), except for high-density lipoprotein cholesterol (HDL) levels. Specifically, significant differences in HDL levels were observed between patients with *HMGCR* rs17238540 TT and *HMGCR* rs17238540 TG genotypes, as well as between patients with *HMGCR* rs17244841 AA and *HMGCR* rs17244841 AT genotypes (Table 4, Table 5 and Table 6).

## 3. Discussion

Cognitive decline is a frequent manifestation in PD patients. As prospective studies show, around one-third of PD patients develop dementia within four to five years [18,19], and the numbers drastically increase in longer observations [20]. Some of the risk factors for PDD were associated with age or disease progression, e.g., advanced age, higher age of onset, longer disease duration, higher Hoehn–Yahr stage, or higher levodopa dosage. However, the risk was also increased in patients with hallucinations, REM sleep behavior disorder, and orthostatic hypotension [18,19,20].

The exact cause of PD remains elusive; multifactorial etiology is the most plausible theory, according to which genetic and environmental factors are likely to contribute. SNPs in the gene encoding HMG-CoA reductase, associated with lower protein activity and LDL synthesis, have recently been investigated in neurodegenerative diseases, including PD [17,21].

It is well known that HMG-CoA reductase participates in the endogenous cholesterol synthesis and its activity may vary in different populations [22]. The impact of high cholesterol levels on PD risk was indicated in Finnish and Korean populations [9,23]. Contrarily, some studies suggest that in elderly and very old people (over 85 years), the correlation between plasma lipid levels and cognitive status may be blurred by age-related cognitive deterioration [24].

Statin treatment increases mRNA expression of the LDL receptor gene, facilitating LDL clearance. Alternative splicing of HMGCR lowered the effect [14], indicating that it may play a potential role in lipid homeostasis and potentially in PD and PDD susceptibility. Numerous studies analyzed genetic factors that may play a crucial role in PD and PDD development, including various pleiotropic SNPs, i.e., in *ABCA7*, *ACT*, *ACE*, *APOE*, and *CD33* genes [16,25,26,27,28]. To our knowledge, *HMGCR* variability has not been analyzed in association with cognitive impairment in PD. In our case–control study, we evaluated possible associations between functional SNPs: rs17244841, rs3846662, and rs17238540 in *HMGCR* gene polymorphisms and PD susceptibility, as well as cognitive impairment: MCI and dementia.

In our study, we did not find any statistically significant differences in *HMGCR* rs17244841, rs3846662, and rs17238540 between PD patients and the control group (*p* = 0.252, *p* = 0.302, and *p* = 0.611, respectively) (Table 2) nor within the PD groups (Table 3). To date, the minor rs17238540 allele has mostly been associated with high blood pressure in response to urinary sodium levels [29,30]. This SNP has also been correlated with stroke risk as an independent factor, in addition to its effect on blood pressure. The G allele carriers have higher systolic blood pressure, and more stroke events [29]. This polymorphism has also been associated with a greater reduction in total cholesterol and LDL levels upon statin treatment [31]. Individuals heterozygous for the G allele of rs17238540 were found to be poor responders to statin therapy in terms of lowering total cholesterol and triglyceride levels [15]. These findings may confirm that high levels of triglycerides and cholesterol could be risk factors for PD patients, since the dysregulation of lipid homeostasis may contribute to the development of the disease. G allele carriers may not benefit from statin therapy and therefore maintain a high risk of PD. Our findings, i.e., the negative correlation of analyzed genetic variants between this study and control groups, bring into question the possible role of *HMGCR* rs17238540 in PD prevalence. This could be explained by the homogeneity of the Polish population concerning many genes and could differ between distinct ethnic groups [32].

We did not find any correlations between PD patients and the control group for *HMGCR* rs17244841 or between PD-MCI and PDD patients, and we did not find any associations between the lipid parameters and the analyzed SNP. However, we did observe increased HDL levels in heterozygotic carriers of *HMGCR* rs17238540 (TG) and rs17244841 (AT). Due to the low number of representative patients, these results should be interpreted with caution. For *HMGCR* rs3846662 GG vs. AA (*p* = 0.062), we found a borderline significance with reference to HDL levels. Expanding the study group to include lipid parameters could determine this uncertainty.

A large-group study by Benn et al. found that low levels of LDL cholesterol may have a protective effect against the development of Alzheimer’s disease [21]. On the other hand, in the same study, the effect of low LDL levels and *HMGCR* and *PCSK9* polymorphisms on the risk of disorders such as vascular dementia, Alzheimer’s disease, and Parkinson’s disease was not proven.

The present study examined different polymorphisms in the *HMGCR* gene, compared to the work mentioned above. However, similar to the results of Benn et al., we did not find any significant association between the polymorphisms and cognitive decline in PD [21].

However, little is known about the effects of rs3846662 on in vivo cholesterol homeostasis in the brain. *HMGCR* undergoes alternative splicing at exon 13 with the presence of the intronic rs3846662 SNP, which leads to a catalytically inactive protein synthesis. Alternative splicing of *HMGCR* was associated with reduced mRNA upregulation of the LDL-C receptor gene and a weaker statin response [33]. Individuals carrying *HMGCR* rs3846662 minor variant showed a poor response to statin therapy, which resulted in increased blood cholesterol levels [34,35]. On the other hand, Leduc et al. reported sex-related differences in the impact of the rs3846662 polymorphism. The major allele was associated with poorer statin efficacy in women, despite higher mRNA transcription compared to other carriers, but not in men [36]. In our study, we did not observe any correlation between PD patients and the control group with regard to *HMGCR* rs3846662 occurrence; however, we did find significant differences between the tested polymorphism and cognitive impairment in PD patients.

In the present study, the prevalence of the G allele of this polymorphism was significantly different (*p* = 0.041) in the studied groups of PD patients: without cognitive impairment, with MCI and with dementia. It potentially indicates the importance of the G allele in delaying the development of dementia in PD. The opposite results were obtained by Chang et al. in their study on the *HMGCR* polymorphism (rs3846662) [37]. They demonstrated that the A allele has a protective effect against late-onset Alzheimer’s Disease (LOAD). Potentially, this can be explained by differences in the genetic background of dementia between patients with PD and LOAD. Similar results were also presented by Leduc et al., who indicated that the major A allele variant of rs3846662 acts as a protective factor and delays the onset of AD [17]. The AA rs3846662 genotype was shown to be strongly protective against AD, especially in women, and to decrease MCI conversion to AD [38].

## 4. Materials and Methods

### 4.1. Study Subjects

This study included 306 Caucasian patients (169 males and 137 females), aged 35–89 years (64.45  ±  9.41), from two regional centers in Poland (Gdańsk in Pomerania voivodeship and Szczecin in Westpomerania voivodeship). The subjects were diagnosed with idiopathic PD according to criteria of the UK Parkinson’s Disease Society Brain Bank clinical diagnostic [39]. The exclusion criteria included clinical symptoms suggesting secondary causes of Parkinsonian syndrome (vascular and drug-induced), features suggestive of atypical Parkinsonian syndromes (multiple system atrophy, progressive supranuclear palsy, and corticobasal syndrome) or history of cardiovascular disease (e.g., stroke and heart failure). Basic anthropometric and lipid level information were collected from all study subjects. Written informed consent was obtained before participating in this study. Enrolled PD patients did not receive statin therapy.

The control group consisted of 249 (age 65.11 ± 9.27 years) healthy individuals randomly selected from the same geographical region as study group. Control subjects’ inclusion criteria are as follows: no parkinsonian symptoms, no history of stroke, and no hepatic or renal dysfunction. The study protocol was approved by the relevant local ethics committee (The Bioethics Committee of the Pomeranian Medical University, KB-0012/151/15) and consisted of each participant donating one milliliter peripheral blood samples via venipuncture during routine medical check-ups, during which BMI was determined. After material collection, the blood samples were stored at −80 °C until isolation.

Patients with PD were divided into three subgroups based on the neuropsychological assessment described below: PD patients without MCI or dementia (PD-non-cognitive impairment, PD-NCI, *n*  =  79), PD patients with MCI (PD-MCI, *n*  =  142), and PD patients with dementia (PDD, *n*  =  85). Clinical and demographic data were obtained through semi-structured interviews and medical documentation.

### 4.2. Neurological Examination

Evaluation of the neurological condition of patients was carried out to achieve two main goals: to confirm the PD diagnosis and exclude any suggestive symptoms of atypical or symptomatic cases. The examinations included the Unified Parkinson’s Disease Rating Scale (UPDRS; part II–IV), the Hoehn–Yahr staging and the Schwab–England activities of daily living scale. All of the evaluations were followed by MRI imaging to exclude other etiologies.

### 4.3. Neuropsychological Assessment

All assessments were conducted by a psychologist expert; the examination procedures and standards were established before the study onset. The patients were examined in the ‘on state’. As a screening tool the Mini-Mental State Examination (MMSE) test was used. Neuropsychological examinations included: the Wechsler Adult Intelligence Scale-Revised (WAIS-R), the Rey Auditory Verbal Learning Test (RAVLT), the Benton Visual Retention Test (BVRT), the Trail Making Test (TMT), the Rey–Osterrieth Complex Figure Test (ROCF), the Verbal Fluency Test, and the Wisconsin Card Sorting Test (WCST). To assess mood disturbances the Beck Depression Inventory Test (BDI) was used. In addition, all patients were examined by means of Parkinson’s Disease-Cognitive Rating Scale (PDCRS). MCI and dementia were defined according to criteria set by Litvan et al. and by Emre et al. [40,41].

### 4.4. Genetic Study

Peripheral venous blood samples were collected from each study subject (tubes containing EDTA) during routine check-ups. Genomic DNA was extracted using the Genomic Mini AX Blood SPIN kit (A&A Biotechnology, Gdańsk, Poland). DNA concentration was measured using DeNovix DS11 FX+ spectrophotometer (Wilmington, DE, USA) and diluted to 20 ng/mL. To determine polymorphisms in the 3-Hydroxy-3-Methylglutaryl-CoA reductase gene (*HMGCR*: rs17238540, rs17244841 and rs3846662), real-time PCR was performed using pre-validated TaqMan allelic discrimination assays (Applied Biosystems, catalogue number 4351379, Waltham, MA, USA) on a ViiA7 Real-Time PCR System (Applied Biosystems, Waltham, MA, USA) (Supplementary Table S1).

### 4.5. Statistical Analysis

Genotype distributions with the Hardy–Weinberg equilibrium were assessed using the χ2 test. Genotype case–control analyses between the study groups were performed using the χ2 test or Fisher exact test. Odds ratios (OR) and 95% confidence intervals (95% CI) were calculated using continuity-corrected Wald interval. For demographic and clinical data, the alignment with normal distribution was tested by means of the Shapiro–Wilk test, and further analyses were performed by means of a one-way parametric ANOVA test or one-way non-parametric ANOVA test (Kruskal–Wallis test). Analyses were performed using Statistica ver. 13.2 software (TIBCO Software Inc., Tulsa, OK, USA). Statistical significance was set at *p*-value ≤ 0.05.

## 5. Conclusions

In the present study, we did not observe any connection between the genetic variations in HMGCR rs17244841 and the development of mild cognitive impairment or dementia in individuals with Parkinson’s disease. We found a correlation between the rs17238540 genotype and PD susceptibility, as well as a minor association between rs3846662 and cognitive status in PD patients. Two-sided analysis of these groups did not reveal any significance. The HMGCR rs17238540 and rs17244841 heterozygotic variants may influence serum HDL levels. However, this study should be replicated in a larger population.

## 6. Study Limitations

A larger sample size should be tested to confirm our observations. As the dementia prevalence in PD patients increases with age, it is possible that during the follow-up, MCI or dementia could affect participants who were cognitively intact at the baseline. We did not match groups according to comorbidities such as arterial hypertension, diabetes mellitus, dyslipidemia, and other vascular risk factors that could influence the diagnosis of cognitive decline. Observed genotypes and allele deviations could differ in a larger sample or in a group of participants from more than two centers or in an older population. It is also possible that the genetic variability in the studied material from peripheral blood may be different in local tissues, i.e., in the brain, thus not reflecting the actual correlation between *HMGCR* gene polymorphisms and cognitive impairment. Our study is the first to extensively analyze the genetic variation in *HMGCR* with regard to PD, cognitive decline, and dementia with reference to lipid parameters. In future research, we will also analyze lipid parameters in the control group and increase the study group with regard to *HMGCR* rs17238540 and rs17244841 to determine their role in HDL lipid levels.

## Figures and Tables

**Table 1:** Demographic and clinical characteristics of PD patients (without cognitive impairment, with mild cognitive impairment and patients with Parkinson’s disease dementia) and control group.

*p*-Value ^a^	*p*-Value ^b^	Control Group	PDD	PD-MCI	PD-NCI	PD	Demographic and Clinical Data	
		(*n* = 249)	(*n* = 85)	(*n* = 142)	(*n* = 79)	(*n* = 306)		
0.593 *	<0.001 *	76/173	49/36	74/68	46/33	169/137		Sex (M/F)
<0.001 ^#^	0.261 ^&^	65.11 ± 9.27	68.60 ± 8.32	62.84 ± 9.04	62.90 ± 9.91	64.45 ± 9.41	[mean ± SD]	Age [years]
		25–83	35–85	39–87	43–89	35–89	[range]	
<0.001 ^#^			59.61 ± 9.61	56.16 ± 10.66	56.54 ± 10.54	57.22 ± 10.42	[mean ± SD]	Age at disease onset [years]
			29–77	28–80	37–87	28–87	[range]	
0.002 ^&^			8.94 ± 5.62	6.70 ± 4.94	6.29 ± 4.63	7.21 ± 5.16	[mean ± SD]	Disease duration
			0.5–24	1–21	0.5–21	0.5–24	[range]	
			(*n* = 80)	(*n* = 131)	(*n* = 71)	(*n* = 282)		
<0.001 ^&^			39.96 ± 22.36	28.46 ± 15.75	21.85 ± 12.09	30.06 ± 18.38	[mean ± SD]	UPDRS (part II–IV) score
			4–101	2–80	1–54	1–101	[range]	
			(*n* = 83)	(*n* = 141)	(*n* = 77)	(*n* = 301)		
0.0005 ^&^		948.25 ± 493.17		782.82 ± 493.84	694.25 ± 391.55	805.78 ± 477.69	[mean ± SD]	Daily L-dopa dosage [mg]
			150–2567	150–1995	150–1750	150–2567	[range]	

PD-NCI: Parkinson’s disease patients without cognitive impairment; PD-MCI: Parkinson’s disease patients with mild cognitive impairment; PDD: Parkinson’s disease dementia patients; UPDRS: Unified Parkinson’s Disease Rating Scale (part II–IV); *p* values calculated by means of *: χ2 test; ^#^: one-way parametric ANOVA test; ^&^: one-way non-parametric ANOVA test (Kruskal–Wallis test); ^a^: PD-NCI vs. PD-MCI vs. PDD group. ^b^: PD vs. control group.

**Table 2:** Distribution of HMGCR genotypes in study group and control group.

OR (95% CI)	*p*-Value ^a^		*p*-Value ^b^	Control Group (*n* = 249)		PD Patients (*n* = 306)		.
				%	*n*	%	*n*	
								***HMGCR* rs17244841**
								**genotype**
-	1	AA + AT vs. TT	0.252	94.38%	235	96.41%	295	AA
1.60 (0.71–3.59)	0.31	AA vs. AT + TT		5.62%	14	3.59%	11	AT
-	1	AA vs. TT		0%	0	0%	0	TT
	1	AT vs. TT						
1.60 (0.71–3.59)	0.31	AA vs. AT						
								***HMGCR* rs17244841**
								**allele**
				97.19%	484	98.20%	601	A
1.58 (0.71–3.51)	0.31	A vs. T		2.81%	14	1.80%	11	T
								***HMGCR* rs3846662**
								**genotype**
0.72 (0.48–1.09)	0.14	AA + AG vs. GG	0.302	32.26%	80	29.74%	91	AA
0.89 (0.62–1.28)	0.58	AA vs. AG + GG		49.60%	123	46.73%	143	AG
0.71 (0.44–1.15)	0.18	AA vs. GG		18.14%	45	23.53%	72	GG
0.73 (0.47–1.13)	0.18	AG vs. GG						
0.98 (0.67–1.44)	0.92	AA vs. AG						
								***HMGCR* rs3846662**
								**allele**
				57.06%	283	53.10%	325	A
0.85 (0.67–1.08)	0.2	A vs. G		42.94%	213	46.90%	287	G
								***HMGCR* rs17238540**
								**genotype**
0.71 (0.25–1.97)	0.6	GG + GT vs. TT	0.611	0.80%	2	0.98%	3	GG
1.22 (0.20–7.38)	1	GG vs. GT + TT		2.41%	6	1.31%	4	GT
1.21 (0.20–7.29)	1	GG vs. TT		96.79%	241	97.71%	299	TT
0.54 (0.15–1.93)	0.36	GT vs. TT						
2.25 (0.25–20.13)	0.61	GG vs. GT						
								***HMGCR* rs17238540**
								**allele**
				2.01%	10	1.63%	10	G
0.81 (0.34–1.96)	0.66	G vs. T		97.99%	488	98.37%	602	T

^a^: Fisher exact test; ^b^: χ2 test; *HMGCR* rs17244841, HWE: PD group *p* = 0.75, control group *p* = 0.65; *HMGCR* rs3846662, HWE: PD group *p* = 0.28, control group *p* = 0.85; *HMGCR* rs17238540, HWE: PD group *p* < 0.01, control group *p* < 0.01.

**Table 3:** Frequencies of analyzed *HMGCR* polymorphisms in PD patients without cognitive impairment, with mild cognitive impairment and dementia.

*p*-Value ^#^	PDD *n* = 65 (%)	PD-MCI *n* = 122 (%)	PD-NCI *n* = 67 (%)	Genotype/Allele	Polymorphism
0.76	63 (96.9)	118 (96.7)	66 (98.5)	AA	*HMGCR* rs17244841: A > T
	2 (3.1)	4 (3.3)	1 (1.5)	AT	
	0 (0.0)	0 (0.0)	0 (0.0)	TT	
0.76	2	4	1	AT + TT	
0.763	(0.8)	(1.6)	(1.5)	MAF (T%)	
***p*-Value ^#^**	**PDD** ***n* = 65 (%)**	**PD-MCI *n* = 122 (%)**	**PD-NCI *n* = 68 (%)**		
0.041	26 (40.0)	36 (29.5)	16 (23.5)	AA	*HMGCR rs3846662: A > G*
	27 (41.5)	64 (52.5)	29 (42.7)	AG	
	12 (18.5)	22 (18.0)	23 (33.8)	GG	
0.112	39	86	52	AG + GG	
0.026	MAF (G%)	(55.15)	(44.26)	(39.23)	
***p*-Value ^#^**	**PDD** ***n* = 65 (%)**	**PD-MCI *n* = 122 (%)**	**PD-NCI *n* = 68 (%)**		
0.19	0 (0.0)	3 (2.5)	0 (0.0)	GG	*HMGCR* rs17238540: T > G
	0 (0.0)	0 (0.0)	0 (0.0)	GT	
	65 (100.0)	119 (97.5)	68 (100.0)	TT	
0.19	0	3	0	GT + GG	
0.037	(0.0)	(2.46)	(0.0)	MAF (G%)	

PD-NCI: Parkinson’s disease patients without cognitive impairment; PD-MCI: Parkinson’s disease patients with mild cognitive impairment; PDD: Parkinson’s disease dementia patients; MAF: minor allele frequency; ^#^: X^2^ test.

**Table 4:** Associations between lipid parameters and *HMGCR* rs17238540 genotypes.

*HMGCR* rs17238540					Parameters
TT vs. TG	TG		TT		
*p*-Value	Mean ± SD	*n*	Mean ± SD	*n*	
0.957 *	65.67 ± 4.93	3	65.90 ± 7.27	69	Age [years]
0.141*	22.77 ± 1.30	3	26.29 ± 4.06	69	BMI [kg/m^2^]
0.130 *	221.00 ± 40.29	3	187.38 ± 37.14	69	CH [mg/dL]
0.035 ^#^	84.47 ± 21.73	3	56.79 ± 13.92	69	HDL [mg/dL]
0.745 *	117.67 ± 31.01	3	111.58 ± 31.64	69	LDL [mg/dL]
0.944 ^#^	94.00 ± 49.76	3	99.52 ± 35.11	69	TG [mg/dL]

^#^—Mann–Whitney U test; *—*t*-test; BMI—body mass index; CH—total cholesterol in serum; HDL—high-density lipoprotein cholesterol in serum; LDL—low-density lipoprotein cholesterol in serum; TG—triacylglycerols in serum.

**Table 5:** Associations between lipid parameters and *HMGCR* rs17244841 genotypes.

*HMGCR* rs17244841					Parameters
AA vs. AT	AT		AA		
*p*-Value	Mean ± SD	*n*	Mean ± SD	*n*	
0.957 *	65.67 ± 4.93	3	65.90 ± 7.27	79	Age [years]
0.141 *	22.77 ± 1.30	3	26.29 ± 4.06	79	BMI [kg/m^2^]
0.130 *	221.00 ± 40.29	3	187.38 ± 37.14	79	CH [mg/dL]
0.035 ^#^	84.47 ± 21.73	3	56.79 ± 13.92	79	HDL [mg/dL]
0.745 *	117.67 ± 31.01	3	111.58 ± 31.64	79	LDL [mg/dL]
0.944 ^#^	94.00 ± 49.76	3	99.52 ± 35.11	79	TG [mg/dL]

^#^—Mann–Whitney U test; *—*t*-test; BMI—body mass index; CH—total cholesterol in serum; HDL—high-density lipoprotein cholesterol in serum; LDL—low-density lipoprotein cholesterol in serum; TG—triacylglycerols in serum.

**Table 6:** Associations between lipid parameters and *HMGCR* rs3846662 genotypes.

			*HMGCR* rs3846662 Genotype								Parameters
AA + GA vs. GG	GG + GA vs.	GG vs. AA	AA vs. GA	GG vs. GA	AA		GA		GG		
	AA										
*p*-Value ^&^					Mean ± SD	*n*	Mean ± SD	*n*	Mean ± SD	*n*	
0.91	0.888	0.986	0.806	0.9	65.36 ± 5.87	11	66.14 ± 6.86	37	65.75 ± 8.33	24	Age [years]
0.667	0.348	0.466	0.339	0.825	26.99 ± 3.46	11	26.07 ± 3.67	37	25.87 ± 4.87	24	BMI [kg/m^2^]
0.807	0.981	0.972	1	0.779	188.71 ± 18.06	11	190.80 ± 38.81	37	185.69 ± 42.96	24	CH [mg/dL]
0.053	0.197	0.062	0.469	0.108	63.62 ± 16.96	11	59.16 ± 14.97	37	53.47 ± 13.98	24	HDL [mg/dL]
0.914	0.32	0.67	0.211	0.751	105.90 ± 13.51	11	114.45 ± 33.06	37	110.54 ± 35.05	24	LDL [mg/dL]
0.256	0.839	0.546	0.932	0.252	95.44 ± 22.08	11	95.72 ± 35.64	37	106.55 ± 39.76	24	TG [mg/dL]

^&^—Mann–Whitney U test; BMI—body mass index; CH—total cholesterol in serum; HDL—high-density lipoprotein cholesterol in serum; LDL—low-density lipoprotein cholesterol in serum; TG—triacylglycerols in serum.

## Data Availability

The data that support the findings of this study, except for patients’ identifiers, are available from the corresponding author upon reasonable request.

## Supplementary Materials

The following supporting information can be downloaded at https://www.mdpi.com/article/10.3390/ijms25168964/s1, Table S1: TaqMan^®^ Assays for Real-Time PCR Reaction.
