# Supplementary material for: Genetic Polymorphisms in the HMGCR Gene and Associations with Cognitive Decline in Parkinson’s Disease Patients
Source: Int J Mol Sci. 2024 Aug 17;25(16):8964. doi: 10.3390/ijms25168964 (PMC11354436; doi:10.3390/ijms25168964)
Supplement: Supplementary file 1 [file ijms-25-08964-s001.zip › ijms-3113441-supplementary.pdf]

Table S1. TaqMan® assays for real-time PCR reaction.

SNP	gene name	gene symbol	SNP location	nucleotide change	TaqMan Assay ID
rs17238540	3-hydroxy-3-methylglutaryl-CoA reductase	*HMGCR*	5q13.3	G>A	C__25652066_10
rs17244841				A>G	C__33431917_30
rs3846662				A>G	C___2838669_10
